# Outpatient neurology diagnostic coding: a proposed scheme for standardised implementation

**DOI:** 10.1136/pn-2021-003286

**Published:** 2023-02-20

**Authors:** Fran Biggin, Jo Knight, Rejith Dayanandan, Anthony Marson, Martin Wilson, Arani Nitkunan, David Rog, Christopher Kipps, Catherine Mummery, Adrian Williams, Hedley C A Emsley

**Affiliations:** 1 Lancaster Medical School, Lancaster University, Lancaster, UK; 2 Department of Neurology, Lancashire Teaching Hospitals NHS Foundation Trust, Preston, UK; 3 Department of Neurology, The Walton Centre NHS Foundation Trust, Liverpool, UK; 4 Department of Molecular and Clinical Pharmacology, University of Liverpool, Liverpool, UK; 5 Neurology Department, St George's Hospital, London, UK; 6 Manchester Centre for Clinical Neurosciences, Salford Royal NHS Foundation Trust, Salford, UK; 7 Neurology Department, University Hospital Southampton NHS Foundation Trust, Southampton, UK; 8 Dementia Research Centre, University College Hospitals NHS Foundation Trust, London, UK; 9 Division of Neurosciences, University of Birmingham, Birmingham, UK

**Keywords:** CLINICAL NEUROLOGY

## Abstract

Clinical coding uses a classification system to assign standard codes to clinical terms and so facilitates good clinical practice through audit, service design and research. However, despite clinical coding being mandatory for inpatient activity, this is often not so for outpatient services, where most neurological care is delivered. Recent reports by the UK National Neurosciences Advisory Group and NHS England’s ‘Getting It Right First Time’ initiative recommend implementing outpatient coding. The UK currently has no standardised system for outpatient neurology diagnostic coding. However, most new attendances at general neurology clinics appear to be classifiable with a limited number of diagnostic terms. We present the rationale for diagnostic coding and its benefits, and the need for clinical engagement to develop a system that is pragmatic, quick and easy to use. We outline a scheme developed in the UK that could be used elsewhere.

## Introduction

Clinical coding is the assignment of standard codes to clinical terms using a classification system. Having an accurate description of outpatient activity coded by diagnosis, rather than simply the number of patients seen, would help to understand how neurology outpatient clinics are used and this would inform service design. Outpatient coding has the potential to improve neurology services (see box 1), for example, to anticipate the necessary capacity to develop headache pathways, or to ensure sufficient specialist nursing or advanced practitioner support.

Box 1 **Benefits of outpatient coding for patients, clinicians and neurology services**

- Allowing more effective use of available resources, thus improving patient access and care. Improved access to neurology outpatient clinics could prevent hospital admission and improve clinical outcomes.
- Improving understanding of the service being delivered. Examples include the proportion of patients with multiple sclerosis receiving disease-modifying therapies or the frequency of brain imaging for people with headache.
- Paving the way for monitoring of clinical outcomes, for example, measurable changes in health, function or quality of life as a result of clinical care. Review of clinical outcomes establishes standards against which clinical practice can be continuously improved.
- Potentially standardising and so enabling closer working with other clinicians and services, service planning, audit and research.
- Opportunity for understanding disparities in neurological care, for example, through linkage with patient demographic and Index of Multiple Deprivation data.
- Outpatient neurology diagnostic coding would inform healthcare planning and resource allocation, as illustrated by the recent study of the burden of neurological disorders across the USA.^10^

Clinical coding requires a reliable, robust system to capture the diagnoses of patients seen, as well as when they were seen, by whom and where.

Internationally, clinical coding is used to record clinical activity and for billing. Billing systems vary around the world, capturing data relevant to the payment system, generally focusing on the type of activity rather than the clinical diagnosis. There is variable central collation of clinical activity and diagnostic codes.

In the UK, inpatient admissions in the National Health Service (NHS) are coded by diagnosis (see box 2) but hospitals and primary care use different coding systems. Most neurology care is delivered in outpatient clinics where there is no mandatory coding of diagnosis.

Box 2 **UK inpatient diagnostic coding**

In the UK, National Health Service (NHS) inpatient data are assigned Healthcare Resource Group codes based on procedure codes and International Classification of Diseases 10th Revision (ICD-10 diagnosis codes, enabling hospitals to be reimbursed for activity. Coding data from inpatient records contribute to commissioning datasets, which are sent by the hospital to the Secondary Uses Service (SUS), an external national data warehouse hosted by NHS Digital. Hospital episode statistics data are derived from cleaned SUS data extracts, and used for a range of analytics, planning services, monitoring and payment.

The coding is done by non-medical coders from their analysis of the medical notes and discharge summary.

Diagnostic coding of outpatients is likely to become part of UK clinical commissioning in the future, and may well become mandatory; thus, clinicians should consider engaging with (and so shaping) the process from the outset.

This paper summarises the current situation, describes relevant pilot work, discusses potential barriers and outlines a proposed standardised scheme for implementing outpatient neurology diagnostic coding.

### Outpatient neurology diagnostic coding

In the UK, the National Neuroscience Advisory Group report^1^ and Getting It Right First Time—an NHS improvement programme focused on improving access to care for patients with neurological disorders across England^2^—both recommended developing outpatient coding to support service planning and to enable benchmarking between different neurology services.

We currently have no standardised mechanism for outpatient neurology diagnostic coding. Some centres are adopting local implementation systems, and some individual neurologists keep their own records, but often the only standardised information recorded is whether the outpatient visit was for a new or follow-up appointment. Despite a longstanding recognition of the need for neurology outpatient coding, there has been no appetite for its standardised implementation, seemingly because of workload pressures and lack of administrative support. In short, the process needs to be clinically driven and pragmatic to avoid excessively burdening clinicians.

The COVID-19 pandemic brought the lack of outpatient coding into sharp focus because of the difficulty in risk stratifying patients with neurological conditions.^3^ Data held centrally by NHS Digital were recognised to be inaccurate and incomplete, and so the task of risk stratification was delegated to individual clinicians, who themselves were severely hampered by the lack of outpatient diagnostic coding data.

The clinician-friendly Systematised Nomenclature of Medicine-Clinical Terms (SNOMED CT; see box 3) is the clinical vocabulary with the greatest momentum with respect to direct care and its readiness for use in clinical research. SNOMED CT permits entry of concepts familiar to clinicians, including symptoms, procedures, clinical measurements, diagnoses and medications. Concepts have unique IDs, but SNOMED CT supports synonyms, allowing the same concept to be expressed in multiple ways. The Wales neurology database has demonstrated SNOMED CT can be used to code for neurological practice.^4^ Wardle and Spencer reported key benefits to be: the ability to understand patient cohorts; recording accurate clinician-derived diagnostic information informs clinical services and facilitates epidemiological work. Comparisons of data between individual patients and whole patient cohorts can be made in real time (eg, comparing the clinical course of multiple sclerosis of an individual versus peers while taking disease-modifying therapy). SNOMED CT also offers the flexibility to add functionality, for example, monitoring botulinum toxin administration including structured data capture. SNOMED CT has other important advantages including interoperability and the ability to encode metadata. In the UK, the National Information Board, charged with developing strategic priorities for data and technology in health and care across NHS, public health, clinical science, social care and local government, endorses the move to SNOMED CT as a single clinical terminology to support direct management of care.

Box 3 **The Systematised Nomenclature of medicine-clinical terms (SNOMED CT)**

The SNOMED CT is a medical terminology designed for input into electronic health records. It comprises concepts organised into hierarchies, descriptions linking human readable terms to concepts and relationships linking concepts to related concepts. SNOMED CT is not an alternative to ICD-10. However, SNOMED CT terms are better suited to clinician use, and can be mapped to ICD-10. More widespread clinical use of SNOMED CT should also facilitate its continued development and ensure that it remains up to date and suitable for use in specialist settings such as neurology. SNOMED CT addresses the requirement for robust interoperability between different systems with the use of appropriate information and data standards.

The FAIR (findability, accessibility, interoperability and reusability) principles^11^ aim to make data findable, accessible, interoperable and reusable in order to maximise its usefulness. Outpatient neurology diagnostic coding requires a standardised approach, which individual hospital information technology (IT) systems and end users will need to adopt. This would include assignment of relevant patient identifiers including NHS number (findable), using a standardised protocol to permit retrieval by identifier (accessible), and use SNOMED CT terms (ensuring interoperability and reusability). A 2002 Audit Commission report entitled ‘Data remember: improving the quality of patient-based information in the NHS’^12^ recommended implementation of SNOMED CT, not least because this system permits clinicians to use familiar diagnostic and procedure terms at the point of care. SNOMED CT terms are a mixture of ‘disorder’, ‘finding’ and other term types based on the commonly used clinical nomenclature (eg, headache is a symptom as well as a disorder, epilepsy is a syndrome). Although other administrative systems such as ICD-10 will still be widely used, ICD-10 coding or similar could be generated automatically from SNOMED CT terms, reducing the burden on the end user.

NHS, National Health Service.

The Sentinel Stroke National Audit Programme illustrated the impact of good data, helping to transform stroke care in the NHS. Stroke teams are active in this audit and the related coding and assessments. Similar principles could apply to neurology with clinicians appreciating the benefits of having open and transparent access to their own data, as well as to data from other users. Box 4 shows some practical applications of outpatient neurology diagnostic coding. By demonstrating the value of data collection, clinicians will increasingly see the value of accurate diagnostic coding, and the importance of their taking ownership of it.

Box 4 **Practical applications of outpatient neurology diagnostic coding: some examples**

**Patient safety.**

Despite the widespread lack of outpatient coding and the adverse impact this had on recent COVID-19 risk stratification exercises,^8^ those clinicians with locally held diagnostic category data benefited from more rapid identification of patients with diagnoses likely to be deemed extremely vulnerable (eg, conditions associated with bulbar dysfunction). There are likely to be many similar situations. For example, being able to identify particular patient cohorts rapidly might help where there is a safety alert, or a need to track patients taking a particular medication (such as sodium valproate).

**Disease monitoring to support clinical decision making.**

‘Live’ use of SNOMED CT in Wales^4^ has shown how it is possible to compare individual patient performance with the whole cohort over time, in real time. Thus clinical coding, with filtering (eg, by disease) and linkage to other relevant clinical data (eg, linking data on patients with multiple sclerosis with Expanded Disability Status Scale (EDSS) scores and use of disease-modifying drugs) can compare disease progression or activity and support clinical decision making.

**Addressing capacity and demand: identifying unexpected rates of referral, using headache as an example.**

In a recent retrospective observational study, we prospectively assigned diagnostic categories.^13^ We collected data from a single consultant outpatient neurology clinic and 202 General Practitioner (GP) surgeries across seven clinical commissioning groups in the northwest of England, and identified 388 new referrals for headache. We applied statistical modelling to identify GP surgeries with unexpected rates of referral, thereby permitting relevant targeted intervention, education and/or support.

**Specialised commissioning.**

While most healthcare is planned and arranged locally, NHS England plans specialised treatment services nationally and regionally for people with rare and complex conditions. However, NHS England is currently transitioning to place-based and population-based commissioning. Such a networked approach—enabling complex patients to be seen locally and yet ensuring funding reflects their needs—depends on having accurate diagnostic coding.

NHS, National Health Service; SNOMED CT, Systematised Nomenclature of Medicine-Clinical Terms.

### Outpatient neurology diagnostic coding: how?

A 2006 Royal College of Physicians survey found that 80% of UK clinicians had little or no contact with coding departments^5^; clinical disengagement seems an important contributor to poor data quality.^6 7^

Factors facilitating clinician engagement with outpatient neurology diagnostic coding include speed, simplicity and ease of use. Above all, for successful implementation in a live clinical setting, the time commitment must be minimal and the payback worthwhile. Local implementation requires support by individual Trusts and Health Boards, owing to the diversity of clinical information systems, but should follow a standardised approach that adheres to some basic principles. This will ensure the system is ‘user-friendly’ with the fewest possible steps or ‘clicks’ to assign a code, and minimal time per entry. Our experience shows that this is readily achievable, although by interested clinicians. We need a pragmatic approach, with a focus on the main neurological condition. Where a patient has been coded once, it should be quicker to code at subsequent appointments providing the diagnosis is unchanged.

It is unrealistic to expect to implement a perfect system immediately, enabling complete coding of all patients. Indeed, it would be preferable that a system captures coding for 80% of all outpatients (new and follow-up) using a small number of codes than to continue to fail to capture coding for 100% of patients. Thus, we should order the specified list of core diagnoses according to their frequency, minimising the time spent scrolling/searching. Many clinicians might wish only to enter minimal ‘high level’ diagnostic categories, while others may wish to enter more detailed ‘granular’ diagnostic information. We should facilitate both approaches.

Decisions regarding the level of coding could be made locally, although it will be sensible to prioritise a minimum core dataset (eg, headline diagnostic category) and include this automatically in clinic letters. Clinicians need to retain the option to enter more granular diagnostic data, preferably by using SNOMED-CT (box 3) and that those choosing to do this should not be penalised with extra work—the ‘high level’ category should be assigned automatically. Essentially, we recommend keeping the system as simple and as quick as possible, to avoid any sense of additional clinician burden.

In order to determine a reliable estimate of diagnostic category frequencies, we combined the data from two large neurology referral studies.^8 9^ Table 1 shows the diagnostic categories and frequencies in the two studies, as well as the combined frequencies and combined proportions.

**Table 1:** Diagnostic category frequencies from two large neurology referral studies

Diagnostic category	Biggin *et al* 8 percentage n=1951	Stone *et al* 9 percentage n=3781	Combined percentage n=5732
Headache (all)	19.4	19.2	19.3
Seizure/epilepsy	14.5	13.6	13.9
Psychological/functional	9.7	15.5	13.5
Peripheral nerve/neuromuscular	8.5	10.5	9.8
Movement disorders (all)	9.2	5.9	7.0
Spinal disorders	5.0	6.2	5.8
Multiple sclerosis/demyelination	2.2	6.7	5.1
Syncope/transient loss of consciousness	5.0	4.1	4.4
Stroke (all)	4.7	3.4	3.9
General medical	1.5	2.4	2.1
Dementia	1.0	0.6	0.7
Brain tumour	0.5	0.6	0.5
Muscle	0.5	0.6	0.5
Motor neurone disease	0.4	0.2	0.3
Miscellaneous neurological disorders	13.9	10.4	11.6
No definite neurological diagnosis	4.0	0.0	1.4
Total	1951	3781	5732

This exercise showed that four of the top five diagnostic categories were common to both studies. We could classify 63.5% of new patients’ working diagnoses into these five diagnostic categories. For simplicity, in the proposed scheme shown below (table 2) we have rounded the indicative percentage frequencies to 20% (headache), 15% (psychological/functional), 15% (seizure/epilepsy), 10%–15% (peripheral nerve/neuromuscular), followed by 5%–10% (demyelination/inflammation, spinal degenerative disease, movement disorder) and 5%–25% (other). Table 2, therefore, includes the headline diagnostic categories arranged in order of frequency in a general neurology clinic, acting as a ‘gateway’ to the SNOMED CT terms.

**Table 2:** Proposed coding scheme

Indicative proportion of new outpatient attendances	Headline categories (10 categories, preferred option)	Subcategories (28 categories)	SNOMED CT terms	
20%	Headache	Migraine	Migraine (finding)	(Multiple SNOMED CT terms)
		Idiopathic intracranial hypertension	Benign intracranial hypertension (disorder)	
		Headache (other)	(multiple SNOMED CT terms)	
15%	Functional/psychological disorder	Functional	(multiple SNOMED CT terms)	
		Anxiety	Anxiety (disorder)	(multiple SNOMED CT terms)
		Depression	Depressive disorder (disorder)	(multiple SNOMED CT terms)
15%	Epilepsy/seizure	Epilepsy	Epilepsy (disorder)	(multiple SNOMED CT terms)
		Seizure	Seizure disorder (disorder)	(Multiple SNOMED CT terms Including non-epileptic attack disorder)
10%–15%	Neuromuscular disorder	Peripheral neuropathy	Peripheral nerve disorder (disorder)	(Multiple SNOMED CT terms)
		Myopathy	Disorder of skeletal and/or smooth muscle (disorder)	(Multiple SNOMED CT terms)
		Myasthenia gravis	Myasthenia gravis (disorder)	(multiple SNOMED CT terms)
5%–10%	Demyelination/inflammation	Multiple sclerosis	Multiple sclerosis (disorder)	(Multiple SNOMED CT terms)
		Other CNS demyelination/inflammation	Demyelinating disorder of the CNS (disorder)	(Multiple SNOMED CT terms)
5%–10%	Spinal degenerative disease	Spinal degenerative disease	Degeneration of spine (disorder)	(Multiple SNOMED CT terms)
5%–10%	Movement disorder	Parkinsonism	Parkinsonism (disorder)	(Multiple SNOMED CT terms)
		Essential tremor	Essential tremor (disorder)	
		Other movement disorder	Movement disorder (disorder)	(Multiple SNOMED CT terms)
5%–25%	Other	Ataxia	Ataxia (finding)	(Multiple SNOMED CT terms)
		Cerebrovascular disease	Cerebrovascular disease (disorder)	(Multiple SNOMED CT terms)
		Cranial nerve palsy	(Multiple SNOMED CT terms)	
		Dementia	Dementia (disorder)	(Multiple SNOMED CT terms)
		Faints/blackouts	(multiple SNOMED CT terms)	
		Traumatic brain injury	Traumatic brain injury (disorder)	(Multiple SNOMED CT terms)
		Sleep disorder	(multiple SNOMED CT terms)	
		Encephalopathy	(multiple SNOMED CT terms)	
		Other	(multiple SNOMED CT terms)	
	Suspected neurological diagnosis	Suspected neurological diagnosis		
	No definite neurological diagnosis made	Symptoms	(Multiple SNOMED CT terms) for example, dizziness, diplopia, multiple symptoms, sensory symptoms, visual disturbance, weakness	
	Not coded			

SNOMED CT, Systematised Nomenclature of Medicine-Clinical Terms.

Local implementation must allow the automatic population of headline (super-ordinate) diagnostic categories where users choose to enter SNOMED CT terms (eg, ‘SUNCT’ would automatically be assigned to ‘headache’ without the clinician making additional steps). Full implementation of the proposed scheme for coding would involve assignment of SNOMED CT terms, thus minimising the proportion of conditions assigned to ‘other’. Inevitably, clinicians will vary in their degree of granularity during routine coding.

During prospective paper-based piloting of this approach locally during the COVID-19 pandemic, we benefited from feedback as well as peer engagement and iterative discussion as it developed. This pragmatic approach balances simplicity (for those wishing only to enter the highest level diagnostic categories—perhaps the only element that would be mandated) with the scope for additional diagnostic granularity where desired, indeed as far as SNOMED CT permits.

Early feedback to clinicians, for example, through dashboards, will be important to maintain motivation and interest. Greater use of SNOMED CT by neurologists should encourage ongoing development of the system. We have included three case studies based on experiences from early implementation (see online supplemental appendix).

10.1136/pn-2021-003286.supp1 Supplementary data

## Conclusion

Outpatient neurology diagnostic coding will provide opportunities to improve delivery of neurological services. Coding is best led by clinicians, and needs to be quick, simple and pragmatic. Our proposed scheme takes account of diagnostic category frequency. Local implementation should permit the clinician to identify only the ‘headline’ diagnostic category if they wish, and perhaps this should be the only mandatory element. However, clinicians must have the option to enter more granular diagnostic data using SNOMED CT terms. The use of SNOMED CT should promote engagement, data completeness, consistency, accuracy and permit adherence to FAIR principles.

The process needs to be clinically led, and the data openly available. Maximising clinical engagement in the process of outpatient neurology coding will depend on implementation that allows for speed, simplicity and ease of use.

**Key points**

- Outpatient diagnostic coding has the potential to improve service delivery and patient care.
- Successful implementation requires the process to be pragmatic and quick.
- A standardised approach will enhance the impact of coding.
- Clinical engagement will be crucial to the success of outpatient neurology diagnostic coding.

**Further reading**

- National Neurosciences Advisory Group (2021) Lessons learnt from the COVID-19 pandemic. Priorities in care for people with neurological conditions after the pandemic. https://www.neural.org.uk/wp-content/uploads/2021/04/Lessons-learnt-from-the-COVID-19-pandemic-Priorities-in-care-for-people-with-neurological-conditions-A-report-by-the-National-Neurosciences-Advisory-Group-NNAG-April-2021.pdf
- Getting It Right First Time (2021) Neurology GIRFT Programme National Specialty Report. https://www.gettingitrightfirsttime.co.uk/medical-specialties/neurology/
- Kemp M, Biggin F, Dayanandan R, Knight J, Emsley HCA. COVID-19 exposes the urgent need for coding of outpatient neurology episodes. BMJ Neurol Open. 2020 Aug 11;2 (2):e000080. doi: 10.1136/bmjno-2020–0 00 080. PMID: 33681802; PMCID: PMC7903171.

## Data Availability

No data are available.
