# Supplementary material for: Outpatient neurology diagnostic coding: a proposed scheme for standardised implementation
Source: Pract Neurol. 2023 Feb 20;23(4):317–22. doi: 10.1136/pn-2021-003286 (PMC10423506; doi:10.1136/pn-2021-003286)
Supplement: Supplementary data [file pn-2021-003286supp001.pdf]

Appendix

Case studies

(1) The Walton Centre NHS Foundation Trust (WCFT), a regional neurosciences centre serving a 3.5 million population across Merseyside, Cheshire, North Wales and the Isle of Man.

At WCFT, in-house software that is used for the majority of the EPR, made the process of developing and implementing outpatient clinical coding system easier. This was introduced approximately 5 years ago. In particular this allowed clinical input into the software from the outset so that coding was embedded within the overall clinical workflow as far as possible.

The layout includes all specialities including 'general neurology' and these sections are collapsed until selected, to keep minimal information on the screen at one time. As few clicks as possible get the user to the right section, which is populated by a list of the most common diagnoses – these are mapped to ICD-10 codes. There is an additional optional section to add relevant co-morbidities, and an option to search for and add a diagnosis if it is not listed. For follow up patients, the form indicates any previously added diagnoses to avoid duplicating work. Completion of the form from start to finish is easily completed within 30 seconds.

(2) Croydon University Hospital – a district general hospital serving an approx. 400,000 population. Croydon has no inpatient neurology beds but strong links to St George's Hospital, the regional neurosciences centre.

CERNER is the electronic patient record system at Croydon University Hospital. Ad hoc coding of neurology outpatients started when CERNER was established in 2013 with the withdrawal of paper records. In 2020 with engaged clinicians driving the process, a favourites folder was created of the common codes used so that coding could be done within 1 minute during a clinic appointment. Liaison between informatics and neurology departments enabled the diagnostic codes to be mapped to headings. A spreadsheet of the data were sent monthly to the department. In April 2021, 75% of all neurology consultant appointments were coded.

(3) Lancashire Teaching Hospitals NHS Foundation Trust (LTH) – which hosts the Lancashire Neurosciences Centre, serving a regional population of approx. 1.6 million in Lancashire and South Cumbria.

Quadramed CPR (QMed) is the electronic patient record system at LTH. Preliminary neurology 'problem lists' were introduced in 2019. In 2022, a working group including three neurologists, the EPR development manager and the director of continuous improvement has overseen the implementation of a pilot system within QMed that mirrors the scheme presented in this paper. This permits assignment of a headline diagnostic category or subcategory in significantly less than 1 minute and is currently achieving wider uptake within the neurology department.
